# Sleep alterations as a predictor of bipolar disorder among offspring of parents with bipolar disorder: a systematic review and meta-analysis

**DOI:** 10.47626/2237-6089-2021-0256

**Published:** 2021-12-10

**Authors:** Kyara Rodrigues de Aguiar, Mariana Dias Cabelleira, Bruno Braga Montezano, Karen Jansen, Taiane de Azevedo Cardoso

**Affiliations:** 1 Programa de Pós-Graduação em Saúde e Comportamento Universidade Católica de Pelotas Pelotas RS Brazil Programa de Pós-Graduação em Saúde e Comportamento, Universidade Católica de Pelotas (UCPel), Pelotas, RS, Brazil.; 2 Universidade Federal do Rio Grande do Sul Programa de Pós-Graduação em Psiquiatria e Ciências do Comportamento Universidade Federal do Rio Grande do Sul Porto Alegre RS Brazil Programa de Pós-Graduação em Psiquiatria e Ciências do Comportamento, Universidade Federal do Rio Grande do Sul (UFRGS), Porto Alegre, RS, Brazil.; 3 Department of Psychiatry and Behavioural Neurosciences McMaster University Hamilton ON Canada Department of Psychiatry and Behavioural Neurosciences, McMaster University, Hamilton, ON, Canada.

**Keywords:** Bipolar disorder, high-risk offspring, sleep disorder

## Abstract

**Introduction:**

Bipolar disorder (BD) has a high heritability rate. Current studies have been dedicated to identifying prodromes of BD in the offspring of parents with BD (BO) and the sleep patterns of these individuals have been considered important factors.

**Objective:**

To describe changes in sleep parameters among offspring of parents with BD when compared to offspring of controls and to identify if changes in parameters and quality of sleep predict the onset of BD among these individuals.

**Methods:**

PubMed, PsycINFO, and Embase were systematically searched with no year or language restrictions, up to August 18, 2020. We searched for a combination of the following search items (“sleep*”) AND (“bipolar disorder*” OR “mania” OR “hypomania” OR “bipolar depression”) AND (“ultra-high risk” OR “high risk” OR “offspring” OR “first degree relatives”).

**Results:**

A total of 10 studies were included in the systematic review and 4 studies were included in the meta-analysis. Our meta-analysis showed that the BO had greater daytime sleepiness as compared to the offspring of control parents. The systematic review indicated that shorter sleep duration, sleep disorders, and other related features can differentiate the two groups. Finally, some sleep patterns such as decreased sleep, difficulty falling asleep, and overall sleep problems might be predictors for the development of BD.

**Conclusion:**

Results from the meta-analysis indicated that BO had greater daytime sleepiness. Qualitative results showed that the offspring of parents with BD have an increased likelihood of experiencing an adverse sleep pattern.

## Introduction

Bipolar disorder (BD) is a chronic mental illness that affects about 2% of the adult population, considering all of the spectrums.^1^ BD has a high heritability rate, and family history of BD is one of the main risk factors for developing the disorder.^2 , 3^ Offspring of parents with BD (BO) are therefore an identifiable high-risk group that may provide relevant information about the course of the emerging disease.

Given the chronic and debilitating nature of the disorder, many recent studies have been dedicated to identifying prodromes of BD in BO, in order to be able to target the prodromes of BD through strategies of prevention and early treatment.^4 - 7^ Diagnosing BD in young people has proven challenging, considering that prodromic symptoms are nonspecific.^8 , 9^ Thus, the sleep patterns of individuals who have parents with BD have been considered an important prodrome of the disorder.^10 - 13^

However, changes in sleep are complex characteristics that can involve many specifics. For example, when sleep is assessed by subjective measures, the instruments present many different domains for sleep assessment,^7 , 13 - 16^ and studies using objective measures are not consistent on how the macrostructure of sleep is affected in BO.^11 , 17 , 18^ In addition, many prior studies considered symptomatic BO in their analyses, making it difficult to determine whether sleep is a prodrome of development of the disease or a consequence of the already established disorder.

In recent years, two systematic reviews have been conducted investigating the link between onset of sleep problems and subsequent development of BD.^19 , 20^ One of those systematic reviews^19^ aimed to describe the current evidence regarding chronotype and circadian rhythm patterns in patients with BD. Forty-two studies were included, involving 3,432 patients with BD. The systematic review concluded that depression was more frequently associated with circadian alterations than euthymia in patients with BD. Mania was also associated with irregular rhythms, although few studies evaluated it. Considering biomarkers, preliminary evidence showed dysregulation of daily levels of melatonin and cortisol in patients with BD. In conclusion, the vast majority of studies showed a disruption of circadian rhythm and an evening preference in patients with BD, independent of mood status. However, the impact on mood status is still unclear.

The other systematic review^20^ described the literature regarding sleep alterations predicting full-blown onset of BD, both in general and according to specific polarities of onset. 16 studies were included, as follows: (1) prospective studies including BO presenting sleep alterations who later developed BD; (2) prospective studies assessing patients with sleep disorders who later developed BD; and (3) retrospective studies including patients with BD who presented sleep alterations before the onset of BD. The systematic review concluded that a decreased need for sleep may precede the onset of illness, especially for a manic episode, while insomnia appears to anticipate either a manic or a depressive episode. The sleep disturbances may frequently take place 1 year or more before the onset of BD, often during childhood or adolescence. Moreover, hypersomnia seems to precede BD episodes. Therefore, sleep alterations frequently happen for a long time before the onset of BD and seem to be specifically related to the polarity of the index episode.

However, none of these systematic reviews included a meta-analysis. Additionally, to the best of our knowledge there are no systematic reviews that have included studies that performed independent analyses of sleep among symptomatic and asymptomatic BO and/or including only studies comparing unaffected BO to offspring of controls. Furthermore, previous reviews were not limited to investigating the sleep patterns in BO, but they also included studies that included first-degree relatives^20^ and individuals with clinical risk (individuals in the general population who developed BD)^19^ in their analyses. Our systematic review will add to the existing literature, considering that we will analyze sleep patterns in a homogeneous and specific population: BO.

Therefore, the objectives of our systematic review and meta-analysis were to describe the changes in sleep parameters (assessed objectively or subjectively) among BO when compared to offspring of parents without BD (CO) (aim 1) and to identify if changes in parameters and quality of sleep predict the onset of BD among offspring of parents with BD (aim 2).

## Review questions

- Do BO have worse sleep quality and/or more sleep disorders in comparison to offspring of parents without BD?
- Do changes in parameters and quality of sleep predict the onset of BD among BO?

## Methods

The Preferred Reporting Items for Systematic Reviews and Meta-analysis (PRISMA)^21^ guidelines were followed for the present review.

### Protocol registration

A protocol for this systematic review was registered prospectively in PROSPERO under the ID CRD42020203654 on September 17, 2020.

### Search strategy

A literature search was conducted on August 18, 2020, with no publication date or language restrictions using the following databases: PubMed, PsycINFO, and Embase. We searched for a combination of the following search items (“sleep*”) AND (“bipolar disorder*” OR “mania” OR “hypomania” OR “bipolar depression”) AND (“ultra-high risk” OR “high risk” OR “offspring” OR “first degree relatives”). The search yielded 587 articles: (Pubmed = 168, PsycINFO = 99, and Embase = 320), with 415 remaining after removal of duplicates.

We used the following inclusion criteria to determine whether an article was relevant to our study: (1) the study should present original data; (2) the study should include BO; (3) the study should include offspring of parents without BD as a non-exposed control group; and (4) both groups of offspring should have been assessed regarding their sleep through an objective or subjective assessment. The exclusion criteria were: (1) reviews and meta-analyses; (2) case reports; (3) conference abstracts; and (4) studies where the entire population of offspring of parents with BD had already been diagnosed with a mood disorder.

Our main outcomes were: (1) sleep quality (e.g. subjective sleep quality, sleep disorders, daytime dysfunction, sleeping medication use); (2) sleep parameters (e.g. sleep efficiency, awakenings during the night, sleep fragmentation index, sleep latency, total sleep time), assessed by objective and/or subjective measures.

**Figure 1:**
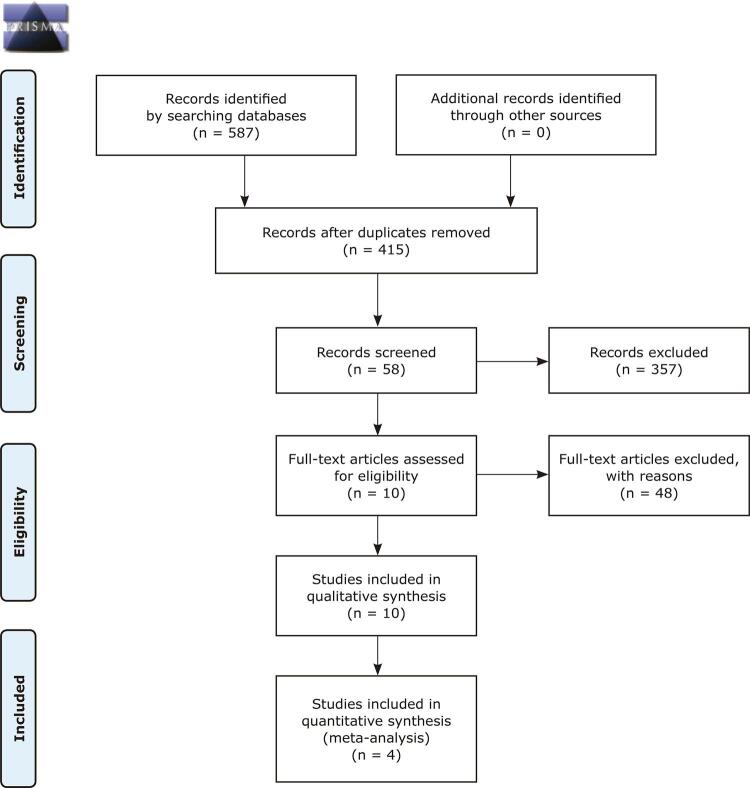
PRISMA 2009 flow diagram. Adapted from Moher et al.23 For more information, visit www.prisma-statement.org.

The studies were assessed by two blinded raters (KRA and MDC) who determined if studies met inclusion criteria. The two raters assessed manuscripts independently using the Rayyan platform^22^ and differences were resolved by consensus among all authors. Initially, the raters screened articles by title and abstract and then by full text. All articles not fulfilling the search criteria were excluded. The details of the search strategy are illustrated in Figure 1 .

### Data extraction

Two researchers (KRA and MDC) conducted the data extraction process. We extracted: authorship, year of publication, country where the study took place, study aims, study design, characteristics of the population, presence of mood symptoms in the sample, assessments, and main results.

### Quality assessment

Each manuscript included was independently assessed by two blinded researchers (KRA and MDC) using the Newcastle-Ottawa Quality Assessment Scale (NOQAS). Disagreements were resolved by consensus among all authors.

### Statistical analysis

Random effects meta-analyses were performed using RevMan 5.3. This was conducted to assess differences in sleep parameters between BO and CO. To achieve this, the means, sample sizes, and standard deviations reported in the studies were used to compute the standardized mean difference or the mean difference in sleep patterns between offspring of parents with BD and offspring of controls. Significance was set at p < 0.05. Cochrane’s Q test was performed to screen for statistical heterogeneity and the Higgins I2 statistic was used to determine the extent of variation between sample estimates with values ranging from 0-100%. If information needed was not reported in the paper, we contacted the authors asking for additional information in order to include the paper in the meta-analysis.

## Results

The literature search yielded 587 studies. Of these, 172 were duplicates and 357 studies were excluded because the titles and abstracts were not relevant to the research topic, leaving 58 potentially eligible studies, the full texts of which were reviewed. At this stage, 48 studies did not meet the inclusion criteria. A total of 10 studies met all inclusion criteria and were included in the systematic review. In addition, we hand-searched the references of the studies included but found no additional studies to include.

Seven of the 10 studies included (5 cohort, 2 cross-sectional studies) had enough data to be included in the systematic review based on the results of subjective measures^4 , 6 , 7 , 15 , 16 , 24 , 25^ and another 3 cross-sectional studies had enough data to be included in the systematic review based on both subjective and objective evaluation.^5 , 11 , 17^ The results will be presented in separate sections, according to the type of measure. Four of the studies provided enough data to be included in the meta-analysis.^5 , 11 , 17 , 25^ There was only enough data to perform the meta-analysis for aim 1. Table 1 shows an overview of the studies included.

**Table 1:** Overview of included studies in the systematic review

Author, year, country	Objective	Study design	Sample characteristics (type, size and characteristics of participants)	Characterization of the sample regarding mood symptoms	Parent’s diagnosis	Assessment instruments for diagnosis of offspring	Sleep assessment instruments for offspring	Main outcomes	Was the hypothesis confirmed?	Study quality
Shaw et al.,^4^ 2005, USA	To identify the frequency and pattern of potentially prodromal symptoms/behaviors for BDI	Cohort, 10 years of follow-up	The sample consisted of 14 families having a BDI parent (numbering 100 children) and 13 matched control families with 110 children. The current bipolar (52 males/58 females) and control (56 each) samples have essentially equal sex distribution and mean ages of 17-18 years	All offspring had no history of BD before selection and no information about depressive symptoms was reported	No explicit information	CARE. The CARE interview schedule was developed by experts in child and adolescent psychiatry and growth and development because the existing standardized interview guides for children were culturally inappropriate for the Amish, having been designed for diagnosis and not fully applicable to well youngsters	CARE	As the BO aged there was a shift from more internalizing symptoms to those that are most often seen as manic behaviors, including decreased sleep and difficulty falling asleep when waking up early in the morning, when compared to the control group	Yes	5/9
Jones et al.,^5^ 2006, UK	To study disorders of cognition, affect, sleep and activity in the development of BD in a sample of high-risk families	Cross-sectional	25 children (13-19 years) of bipolar parents were compared with 22 similar aged children of age and sex matched healthy controls	14 BO had current or lifetime mood diagnoses compared with 2 CO	SCID was administered to the parents to confirm diagnosis according to DSM-IV criteria	SADS – Lifetime version (SADS-L)	Actigraph (Actiwatch; Cambridge Neurotech, Cambridge, UK) on their non-dominant wrist for 7 days PSQI	This study analyzed results of symptomatic and asymptomatic BO between each other and in comparison with the CO. Objective measurements showed that BO went to sleep more quickly and with less fragmentation of sleep. Subjective measurements found more sleep disturbance in BO with the clearest disturbances observed in the affected BO.	Yes	7/9
Singh et al.,^24^ 2008, USA	To examine the relationship between temperament and psychopathology in child offspring of parents with BD	Cross-sectional	Offspring (8-18 years) of parents with bipolar I disorder (BO, n = 31) and demographically similar healthy offspring of parents without any DSM-IV diagnosis (CO, n = 21)	Among the BO, 19 (61%) had at least one mood disorder	SCID – Patient edition (SCID-P)	The Washington University in St. Louis Kiddie SADS (WASH UK-SADS) was administered to BO.^26^ Symptom severity was assessed by the YMRS^27^ and the self-report IDS.^28^	The self-report instrument DOTS-R - including Activity Level–Sleep	BO who do not have a mood disorder have a greater ability to follow the same daily sleep patterns	No. The study compared BO to CO but only found significant differences regarding sleep between BO without mood disorder and BO with mood disorder	7/9
Egeland et al.,^6^ 2012, USA	To identify the pattern and frequency of prodromal symptoms/behaviors associated with onset of BDI disorder during childhood or adolescence	Cohort - 16 years’ follow up	The bipolar sample had 115 children with a BDI parent. The control sample had 106 children of well parents, with and without a positive family history for mood disorders. At the time of recruitment, all children in 10 of the 14 CARE families were pre-school or in school (age 14 and younger)	All offspring had no history of BD before selection and no information about depressive symptoms was reported	No explicit information	CARE	CARE	Decreased sleep, difficulty falling asleep, and early morning awakening emerged as significantly more frequent in children who developed BDI as compared to children who did not develop BDI (prior to age 19) and decreased sleep was included among the best-ranked predictors for children with onset of BDI	Yes	5/9
Levenson et al.,^15^ 2015, USA	To compared sleep and circadian phenotypes among three groups: BO diagnosed with BD, BO without BD at intake and offspring of matched control parents who did not have BD	Cohort	BO diagnosed with BD (BD/BO; n = 47) and without BD (non-BD/BO; n = 386) at intake and CO who did not have BD (n = 301)	Study declared no information about depressive symptoms in BO without BD diagnosis	SCID^29^	K-SADS – Present and Lifetime Version (K-SADS-PL^30^): offspring lifetime psychiatric disorders The SCID was used for diagnosis of non-mood psychiatric disorders among offspring aged 18 or older The K-DRS and the K-MRS were used to evaluate mood disorder diagnosis COBY study.^31^ A diagnosis of BD-NOS was made using operationalized criteria	SSHS	Frequent nighttime awakenings, inadequate sleep, longer time to fall asleep on weekends significantly predict conversion to BD	Yes	7/9
Soehner et al.,^25^ 2016, USA	This study tested associations between sleep duration, reward circuitry function, and mood dysregulation in BO	Cross-sectional	Two groups of participants (9-17 years old) who were not affected by BD were included in this study: 25 BO and 21 age, sex, and IQ-matched CO with non-BD psychopathology	Relative to CO, BO exhibited greater symptoms of positive mood/energy dysregulation (PGBI-10M) and mood lability (CALS-P)	SCID-I^29^	Offspring Axis I psychopathology was assessed using K-SADS – Present and Lifetime Version (K-SADS-PL^32^). To evaluate aspects of mood dysregulation in their children, parents also completed the PGBI-10M to assess positive mood and energy dysregulation in the past six months^33^ and the CALS-P to assess severity of mood lability.^34^ Elevated scores on these mood dysregulation measures (PGBI-10M, CALS-P) have been linked to a BD diagnosis in youth.^34,35^	Modified version of the self-report PSQI - sleep patterns and quality in the prior week	In BO, sleep duration was negatively correlated with PGBI-10M and CALS-P (p = 0.049). In CO, sleep duration did not correlate with CALS-P and there was not a sufficient range of PGBI-10M scores in this group to perform a correlation analysis.	Yes	8/9
Levenson et al.,^16^ 2017, USA	To extend the results of the cross-sectional study by Levenson et al.^15^ (2015) showing that sleep disturbance at baseline can be a prognostic indicator of BD development in high-risk youth using data from baseline and follow-up BIOS assessments, characterizing longitudinal sleep phenotypes in BOP and CO during middle and high school years	Cohort, evaluations every two years	335 BO, 277 CO. Offspring ages 6-18 from each family were included. Community control parents were healthy or diagnosed with non-BD psychiatric disorders, group matched by age, sex, and neighborhood. Half were female (50.2%), mean age at their first sleep assessment was 12.81 years (standard deviation = 2.25) and nearly 36% were at advanced pubertal status	Participants in each sleep group differed only on likelihood of being diagnosed with MDD, with youth in the poor sleep group significantly more likely to have MDD and have a parent with BD	SCID^29^	K-SADS – Present and Lifetime Version (K-SADS-PL): offspring lifetime psychiatric disorders The SCID was used for diagnosis of non-mood psychiatric disorders among offspring aged 18 or older The depression section of the KSADS-P (K-SADS Depression Rating Scale (K-DRS) and the K-SADS Mania Rating Scale (K-MRS) were used to evaluate mood disorder diagnosis COBY study^31,35^: a diagnosis of BD-NOS was made using operationalized criteria	SSHS^37^: 1) good sleepers: very low incidence of sleep deficiencies over the six sleep domains; 2) poor sleepers: moderate to high incidence of sleep deficiencies in nearly all of the sleep domains; 3) variable sleepers: high incidence of weekend-to-weekday sleep variability and low incidence of sleep deficiencies over all other sleep domains	The poor sleep group had more than four times the odds of developing BD as those in the good sleep group. However, differences were not statistically significant.	No. The authors speculated that this was probably due to the small sample size that converted to BD	7/9
Sebela et al.,^11^ 2017, Czech Republic	To extend the knowledge of sleep characteristics in offspring at risk for BD	Cross-sectional	42 BO (mean age 12.5±3.2) and 42 sex and age matched comparison CO	5 cases of bipolar spectrum disorders and 6 cases of depressive spectrum disorders were found in the BO group No cases of bipolar spectrum disorders or depressive spectrum disorders were found in the control group	SADS – Lifetime version (SADS-L)	Kiddie SADS – Present and Lifetime version (KSADS-PL)	Actigraphic device (MotionWatch8, CamNTech, Cambridge, UK) for 14 days MEQ to assess circadian preference PSQ for investigation of childhood sleep related breathing disorders and prominent symptom complexes GBI – Sleep Subscale was used to assess sleep disturbances typical for BD	The subjective BO assessment found increased sleep and average insomnia, regardless of mood and energy levels, and depressed mood with insomnia at the beginning of sleep. In the analysis of the separate items, the BO reported significant worsening in the item depressed mood with insomnia at the beginning. The actigraphic results found a longer sleep latency among BO compared to CO	Yes	8/9
Duffy et al.,^7^ 2019, Canada	To describe the emergent course of BD in BO subgrouped by parental response to lithium prophylaxis	Cohort study, annual evaluations - beginning in 1996	This study included 116 high-risk and 55 control families, contributing a total of 279 high-risk offspring and 87 control subjects. Eligible offspring from identified high-risk and control families were in the age range of 5-25 years at baseline	The study did not report any information about depressive symptoms at baseline, only that BO started showing depressive disorders earlier than the controls.	SADS – Lifetime version (SADS-L)	Kiddie SADS – Present and Lifetime version or the SADS – Lifetime version	Kiddie SADS – Present and Lifetime version or the SADS – Lifetime version	Subliminal sleep symptoms were associated with an increased risk of transition from stage 0, where all offspring are fine but at family risk, to stage 1, where non-mood disorders start to emerge and sleep disorders are present in BO that developed BD	Yes	6/9
Sebela et al.,^17^ 2019, Czech Republic	To evaluate the circadian rhythm of rest activity and the macrostructure of sleep using actigraphy in a sample of unaffected children and adolescent children of bipolar and control parents	Cross-sectional	Child and adolescent BO (n = 43; 21 females; 11.0±3.2 years) and CO (n = 42; 17 females; 11.1±3.4 years) comparable in sex and age	The BO had higher GBI scores for depression and mania	SADS	Kiddie SADS – Present and Lifetime Version Psychometric scales were administered to obtain information on subsyndromal mood dysregulation, anxiety symptoms and the presence of sleep disturbances	Motion Watch 8 (Camntech, Cambridge, UK) actigraph on their nondominant wrist for ≥14 days GBI – Parent Version - the sleep subscale was used to assess sleep disturbances	Through objective measurement, the BO had shorter sleep time, lower sleep efficiency and lower prolongation of time in bed on free days. Through subjective measurement, the BO had higher GBI scores (depression;10-item mania; sleep) than the CO and a significant negative association between the GBI sleep score and sleep efficiency on free days in the child subgroup.	Yes	6/9

BD = bipolar disorder; BD-NOS = bipolar disorder not otherwise specified; BDI = bipolar disorder type I; BIOS = Pittsburgh Bipolar Offspring Study; BO = offspring of parents with BD; BOP = bipolar offspring parents; CALS-P = Child Affective Lability Scale - Parent Report; CARE = Child and Adolescent Research and Evaluation; CO = offspring of parents without BD; COBY = Course and Outcome of Bipolar Youth; DOTS-R = Dimensions of Temperament – Revised; GBI = General Behavior Inventory; IDS = Inventory of Depression Scale; K-DMS = K-SADS Mania Rating Scale; K-DRS = K-SADS Depression Rating Scale; K-SADS = SADS for School-Age Children; MDD = major depressive disorder; MEQ = Morningness/ Eveningness Questionnaire; PGBI-10M = Parent General Behavior Inventory-10 Item Mania Scale; PSQ = Pediatric Sleep Questionnaire; PSQI = Pittsburgh Sleep Quality Index; SADS = Schedule for Affective Disorders and Schizophrenia; SCID = Structured Clinical Interview for DSM-IV Axis I Disorders; SSHS = School Sleep Habits Survey; YMRS = Young Mania Rating Scale.

The quality assessment of the studies included showed that most of the studies (n = 4) scored 7 out of 9, indicating good quality ( Table 1 ).

### Sleep patterns in BO assessed using objective measures: evidence from cross-sectional studies (aim 1)

All studies used Motion Watch 8 as an objective sleep analysis tool, two studies used it for 14 days^11 , 17^ and one for 7 days.^5^ Actigraph data available in the literature are limited and controversial. One study with 43 BO children and adolescents and 42 CO found that the BO had shorter sleep time (p = 0.007), lower prolongation of time in bed on free days (p = 0.046), and lower sleep efficiency (p = 0.01), with a significant negative association between the GBI sleep score and sleep efficiency on free days in the child subgroup (p < 0.05).^17^ Both CO and BO had prolonged sleep time on free days, however, this sleep prolongation was lower in BO and it was more pronounced in older participants (p < 0.001). This study discusses the possibility that BO have dysregulated sleep homeostasis. Additionally, when another study with 42 BO and 42 CO^11^ compared actigraphic results, it found longer sleep latency among the BO compared to the CO, which remained significant even after adjusting for confounding factors (p = 0.048). These data were not significant in the previous study and may be complementing the literature. However, another study^5^ including 25 BO and 22 CO showed results in the opposite direction to those presented so far, indicating that BO went to sleep more quickly (p < 0.05), and with less fragmentation of sleep (p < 0.03). Although BO’s objective sleep seems to be better in this study, their subjective experience was the opposite (this will be reported later).

### Sleep patterns in BO assessed using subjective measures: evidence from cross-sectional studies (aim 1)

Several different instruments are used to assess sleep subjectively (for more information see Table 1 ). The subjective results showed that sleep disorders,^5^ increased sleep, and insomnia^11^ are characteristics that differ between BO and CO. A study including 25 BO and 22 CO reported more sleep disturbances in BO, with the clearest disturbances observed in the affected children of parents with BD.^5^ Post-hoc tests indicated significant differences between affected and non-affected BO (p < 0.001) and between affected BO and unaffected CO (p < 0.001), with greater disturbances among affected BO in both cases. This study shows that even when objective sleep assessment is better in BO, their perception of that sleep was inadequate, suggesting that BD may be partially characterized by a greater sensitivity to circadian interruption. Another study with 31 BO and 21 CO indicated differences between the affected and unaffected BO, but not when compared with CO.^24^ In this study, BO without mood disorders had a greater ability to follow the same daily sleep patterns (p = 0.04). This finding suggests that changes in sleep are a condition of the mood disorder.

Regarding sleep duration in BO, a study including 35 BO and 35 CO found a negative correlation with PGBI-10M (p = 0.031) and CALS-P (p = 0.049).^25^ In CO, sleep duration did not correlate with CALS-P and there was not a sufficient range of PGBI-10M scores in this group to perform a correlation analysis. Another study including 42 BO and 42 CO found that the BO had increased sleep duration (p = 0.04) and average insomnia (p = 0.02) regardless of mood and energy levels, and depressed mood with insomnia at the beginning of sleep (p = 0.04)^11^ according to parental report. In the analysis of separated items, the BO reported significant worsening in the item depressed mood with sleep-onset insomnia. Finally, another study including a sample of 43 BO and 42 controls found that the BO had higher GBI scores (depression p < 0.001; 10-item mania p < 0.001; sleep p = 0.02) than the CO.

### Sleep patterns in BO assessed using subjective measures: evidence from cohort studies (aim 2)

Several different instruments were used to assess sleep subjectively ( Table 1 ). Cohort studies found changes in sleep that may predict the onset of BD, including decreased need for sleep, difficulty falling asleep,^4 , 6^ waking up earlier than usual,^6^ waking up at night, inadequate sleep^15^ and sleep disturbances.^7^ In a 10-year prospective study of prodromal patterns for BD type I among Amish Youth, including 15 families with BD-I and their 110 BO and 13 healthy control families with 112 CO, the risk of developing BD among the BO was 20% higher than the CO.^4^ As the BO got older, there was a shift from more internalizing symptoms to those that are most often seen as manic behaviors, including decreased sleep and difficulty falling asleep when waking up early in the morning (p < 0.05) when compared to the control group. Another study of the same population-based cohort conducted a third wave assessment and had similar findings, indicating difficulty falling asleep (p < 0.01).^6^ The risk of BD rating was significantly higher among the BO compared to the CO (p < 0.01). It was reported that another two new symptoms, decreased sleep and early morning awakening (p < 0.01), emerged as significantly more frequent in children who developed BD-I as compared to children who did not develop BD-I (prior to age 19).

Similarly, a more recent study including BO diagnosed at intake with (BD/BO; n = 47) and without BD (non-BD/BO; n = 386) and CO (n = 301) found that a longer time to fall asleep on weekends (p = 0.031) and frequent nighttime awakenings (p = 0.017) significantly predict conversion to BD.^15^ Additionally, even when controlling for lifelong psychiatric disorders, the BD/BO group reported higher rates of inadequate sleep (p < 0.001) compared to the other groups (non-BD/BO, CO). These analyses suggest that subjective measures of sleep quality (i.e., inadequate sleep, waking up at night) have the greatest effect on the discrimination of groups of offspring since BD/BO were 2.3 or 2.4 times more likely to report inadequate sleep (reported by parents) as compared to the non-BD/BO and CO, respectively. Additionally, another study including 279 high risk BO and 87 matched CO, developed a model of the BD clinical trajectory in BO.^7^ The model starts at stage 0, where all offspring are healthy but at family risk. Subsequently, it progresses to stage 1 where non-mood disorders (e.g., anxiety and sleep disorder) start to emerge, transitioning to minor mood disorders, then major depressive disorder, and lastly to BD. Subliminal sleep symptoms were associated with an increased risk of transition from stage 0 to stage 1 (p = 0.036) after adjusting for other subliminal symptoms in the BO group.

Finally, there was a cohort study that failed to observe the impact of sleep on the development of BD.^16^ This study included 335 offspring of parents with BD and 227 offspring of healthy control parents. The main findings showed that in middle adolescence (ages 14-16), youth in the “poor sleep group” were significantly more likely to have a parent with BD than those in the “variable sleep group” (p < 0.01), while at ages 16-18 youth in the “poor sleep group” were significantly more likely to have a parent with BD than those in the “good sleep group” (p = 0.02).The poor sleep group had more than four times the odds of developing BD as those in the good sleep group (OR = 4.25), however, the differences were not statistically significant. This study failed to demonstrate statistical significance but suggests clinical significance.

### Meta-analysis of studies comparing sleep patterns between BO and CO using subjective measures

*Association between daytime sleepiness and BO^5 , 11^*

**Figure 2:**
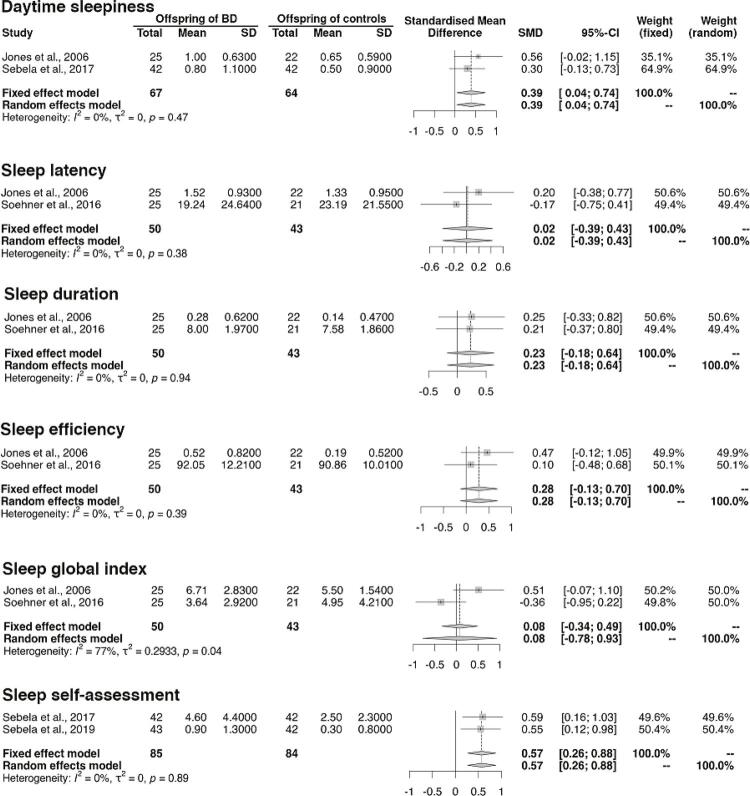
Meta-analysis comparing sleep patterns assessed by subjective measures between offspring of parents with Bipolar Disorder (BD) and offspring of controls.

We found that the standardized mean difference between groups (BO and CO) was 0.39 (95%CI 0.04, 0.74; p = 0.03), indicating higher daytime sleepiness in BO in comparison to CO ( Figure 2 ).

*Association between sleep latency and BO^5 , 26^*

We found that the standardized mean difference between groups was 0.02 (95%CI -0.39, 0.43; p = 0.93), indicating no significant difference in sleep latency between BO and CO ( Figure 2 ).

*Association between sleep duration and BO^5 , 25^*

We found that the standardized mean difference between groups was 0.23 (95%CI -0.18, 0.64; p = 0.27), indicating no significant difference in sleep duration between BO and CO ( Figure 2 ).

*Association between sleep efficiency and BO^5 , 25^*

We found that the standardized mean difference between groups was 0.28 (95%CI -0.13, 0.70; p = 0.17), indicating no significant difference in sleep efficiency between BO and CO ( Figure 2 ).

*Association between sleep global index and BO^5 , 25^*

We found that the standardized mean difference between groups was 0.08 (95%CI -0.70, 0.93; p = 0.86), indicating no significant difference in sleep global index between BO and CO ( Figure 2 ).

*Association between sleep self-assessment and BO^11 , 17^*

We found that the standardized mean difference between groups was 0.57 (95%CI -0.26, 2.60; p = 0.11), indicating no significant difference in sleep self-assessment between BO and CO ( Figure 2 ).

### Meta-analysis of studies comparing sleep patterns between BO and CO using objective measures

*Association between sleep duration and BO^5 , 11 , 17^*

**Figure 3:**
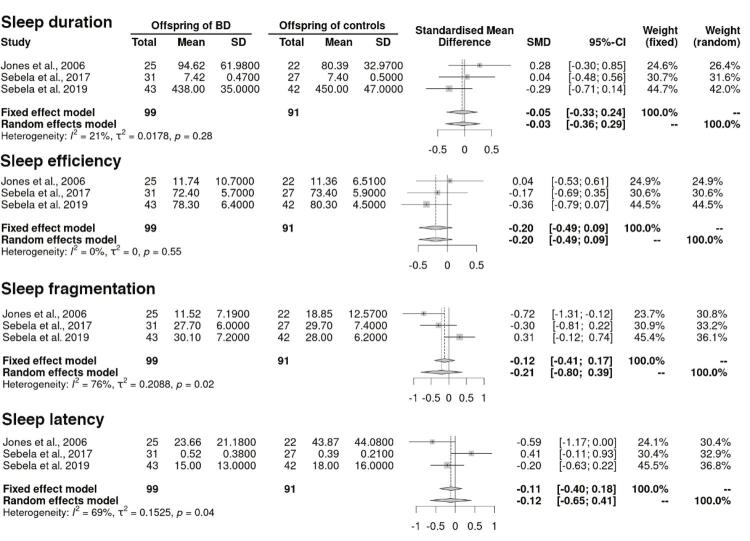
Meta-analysis comparing sleep patterns assessed by objective measures between offspring of parents with Bipolar Disorder (BD) and offspring of controls.

We found that the standardized mean difference between groups was -0.03 (95%CI -0.36, 0.29; p = 0.83), indicating no significant difference in sleep duration between BO and CO ( Figure 3 ).

*Association between sleep efficiency and BO^5 , 11 , 17^*

We found that the standardized mean difference between groups was -0.20 (95%CI -0.49, 0.09; p = 0.17), indicating no significant difference in sleep efficiency between BO and CO ( Figure 3 ).

*Association between sleep fragmentation and BO^5 , 11 , 17^*

We found that the standardized mean difference between groups was -0.21 (95%CI -0.80, 0.39; p = 0.50), indicating no significant difference in sleep fragmentation between BO and CO ( Figure 3 ).

*Association between sleep latency and BO^5 , 11 , 17^*

We found that the standardized mean difference between groups was -0.12 (95%CI -0.65, 0.39; p = 0.41), indicating no significant difference in sleep latency between BO and CO ( Figure 3 ).

## Discussion

Our meta-analysis showed that BO presented higher daytime sleepiness as compared to CO. Additional evidence from the systematic review indicates that shorter sleep duration,^17 , 25^ lower sleep efficiency,^17^ sleep disturbance, insomnia,^11^ lower prolongation of time in bed on free days,^17^ and less fragmentation of sleep^5^ may differentiate the two groups. In addition, our systematic review also shows that decreased sleep,^4 , 6^ difficulty falling asleep,^4 , 6^ early morning awakening,^6^ sleep disorders,^7^ frequent nighttime awakenings, longer time to fall asleep on weekends, and inadequate sleep^15^ might be predictors for BD development.

A previous systematic review^19^ reported evidence regarding the chronotype and circadian rhythm patterns in patients with BD. It concluded that the vast majority of studies showed a disruption of circadian rhythm and an evening preference in patients with BD, independently of mood status, and the authors hypothesized that circadian disturbances may have a role in the pathogenesis of mood disorders. In the current systematic review, our results add to the literature the observation that problems in sleep patterns seem to be a warning sign of manifestation of BD in BO.^4 , 6 , 16^

In addition, another previous systematic review^20^ also reported a relationship in which sleep alterations anticipate full-blown onset of BD. That study^20^ concluded that patients that developed BD may have a decreased need for sleep before BD onset. In this study, insomnia appears to anticipate either a manic or a depressive episode, while hypersomnia seems to be a potential prodrome of onset of a bipolar depressive episode. The previous systematic review included individuals with any first-degree relative with BD, and not only parents with BD. Our systematic review included only BO, to better understand the potential future development of this high-risk population. Our findings showed that sleep disturbances,^7^ decreased sleep,^4 , 6^ difficulty falling asleep,^4 , 6^ early morning awakening,^6^ frequent nighttime awakenings, and inadequate sleep^15^ could anticipate the first onset of BD.

Although BD may present a progressive course,^38^ prodromal symptoms signaling the onset of the disease are still nonspecific.^8 , 9^ The high degree of heritability of the disease^2^ and the results found in our study demonstrate the importance of early diagnosis of BD and emphasize that sleep management may be a relevant strategy to achieve better prognosis in this high-risk population, in which the chances of developing the disease are increased by 9 times.^39^ Changes in sleep and circadian functioning are essential characteristics of the pathophysiology of BD^40^ and interventions involving sleep hygiene should be strongly encouraged as they are malleable problems and intervention may help to delay the progression of the disorder.

Our study selection was restricted to studies including non-affected BO. However, we did not find studies that completely excluded symptomatic BO. We therefore decided to include studies that at least analyzed the symptomatic and asymptomatic BO separately compared to CO. The results of the qualitative analysis targeted those asymptomatic offspring in order to identify if there is a difference in the sleep parameters of BO and CO without the disorder, when both groups of offspring do not present any mood disorders. However, we observed that even studies that did perform this division ended up directing their results towards symptomatic offspring and/or total samples because they found no differences when the offspring were not presenting any mood symptoms (this might be due to the reduced sample of asymptomatic BO). Due to the scarcity of data from asymptomatic BO, our meta-analysis considered the symptomatic and asymptomatic groups together when compared to controls. Thus, these results should be understood taking into account that the presence of depressive symptoms in the BO may bias the results, since changes in sleep may be due to depressive symptoms and not necessarily a prodrome for BD. For this reason, just a few studies (n = 4) were included in the meta-analysis and we were only able to test our hypothesis for objective 1 of the study. It was not possible to verify whether sleep disorders are a prodrome of BD through meta-analysis. However, it is worth mentioning that this is the first study that has aimed to make the target population as homogeneous as possible, disregarding clinical risk or first-degree relatives in general and has carried out a meta-analysis of the existing data.

Taking into consideration that the majority of these studies included BO who had already presented some mood alterations, we were unable to conclude that these impairments and changes in sleep are an early marker of BD. However, BO have an increased likelihood of experiencing an adverse sleep pattern. Therefore, doctors, parents and psychologists should monitor the sleep patterns of this population at high risk of developing BD, since any alteration may signal a greater risk of developing the first BD symptoms. In addition, interventions based on sleep hygiene are encouraged for this population, especially if individuals already have signs of inadequate sleep.

Addressing these research questions was a challenge for us and we believe that it will continue to present a great challenge for researchers in the area, considering the difficulty of access to this population when still asymptomatic, at an early age. However, this is a population that can provide many answers in understanding the development of BD. Thus, future studies should invest in prioritizing the homogeneity of their samples as much as possible with respect to age and to their assessment, prioritizing unaffected children, in the absence of any mood disorder. Furthermore, studies should investigate effective sleep-based interventions for this population.
